# Combining the Pitcher and Lotus Plant: Supericephobic and Superhydrophobic Silicone Films

**DOI:** 10.1002/advs.202520538

**Published:** 2026-02-21

**Authors:** Cliff L. W. Ng, Joshua Ham, Sanpreet Kaur, Clayton W. Schultz, Hua‐Zhong Yu

**Affiliations:** ^1^ Department of Chemistry and 4D Labs Simon Fraser University Burnaby British Columbia Canada; ^2^ Department of Clinical Chemistry Faculty of Allied Health Sciences Chulalongkorn University Bangkok Thailand

**Keywords:** hierarchical nanostructures, liquid‐infused porous surface (SLIPS), nanocontact molding, polydimethylsiloxane (PDMS), superhydrophobicity, supericephobicity

## Abstract

From power lines and airplane wings to wind turbines, many devices and infrastructure would benefit from icephobicity, the ability for a material to shed ice, and thus avoid costly damages which disrupt critical aspects of daily life. Many existing icephobic materials suffer from durability issues simply due to weathering and contamination, which are often addressed by a related but distinct property: superhydrophobicity. Unfortunately, most superhydrophobic surfaces developed to date are not icephobic. Based on bench‐top nanomolding of polydimethylsiloxane (PDMS) with optimized silicone oil content from crystalized polycarbonate (PC) template, we developed hierarchically structured silicone films (as a new class of slippery lubricant‐infused porous surface, SLIPS) that are both superhydrophobic and supericephobic. In doing so, we effectively combine the properties of the lotus and pitcher plant; we explored how the morphology at nano/micrometer scale and the amount of silicone oil in our SLIPS can be tuned to balance wettability and ice shedding to achieve superhydrophobicity and supericephobicity simultaneously, with water contact angle as high as 171.2 ± 1.5° and ice adhesion strength as low as 11.5 ± 2.3 kPa (i.e., superior water repellency and ice shedding capability). While synergistically utilizing the properties of *Nepenthes* and *Nelumbo nucifera*, this new fabrication approach for SLIPS promises tremendous application potentials.

## Graphical abstract

Based on bench‐top nanomolding of polydimethylsiloxane (PDMS) with optimized silicone oil content, we developed hierarchically structured silicone films by essentially combining the properties of the lotus and pitcher plant; particularly we are able to achieve water contact angle as high as 171° and ice adhesion strength as low as 11.5 kPa (i.e., extreme water repellency and ice shedding).

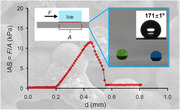

## 1 Introduction

On December 23, 2022, more than 300 flights were cancelled at Vancouver International Airport due to ice buildup on aircrafts [1]. Between midnight and 2 pm, only 51 aircrafts were de‐iced and ready for departure. The situation was a catastrophe for passengers and airlines alike. The usefulness of a passive anti‐icing surface is thus blatantly evident. From helpserving aviation industry to improving wind turbine efficiency, anti‐icing surfaces bring tremendous benefits to human safety, energy conservation, and broadly, the economy. While ice repellency is a sought‐after property for many materials, durable and effective ice anti‐icing surfaces are still hard to find despite years of research and development [2, 3, 4]. Icephobicity is a relatively recent term used in the literature to describe surfaces that demonstrate ice‐repellent properties, typically characterized by the reduced accretion of ice on a surface, as well as a greater ability to shed ice from a surface with minimal external energy input [2, 3, 4].

Icephobic surfaces can be fabricated using various methods, of which many rely on liquid or liquid‐like coatings that enable easy ice detachment [2, 5, 6, 7, 8]. These surfaces are generally “passive”, i.e., they shed ice through surface chemistry, mechanical compliance, or slippage for example. In parallel, recent work has begun to explore materials with icephobic or anti‐icing behavior based on energy‐assisted or hybrid approaches, such as magnetism, electrothermal, and/or photothermal stimulation [9, 10, 11, 12]. While these approaches enable different strategies which may be advantageous in certain areas, passive icephobicity remains attractive, as for certain applications external energy input is impractical or too intensive. Perhaps the most well‐established surfaces used for passive icephobicity are slippery liquid‐infused porous surface(s) (SLIPS), which are typically composed of a porous matrix infused with a lubricant that forms a liquid layer on the surface [13, 14, 15]. However, one of the primary challenges encountered with SLIPS is the depletion of lubricant from the surface [16]. Their lubricants can often be easily lost through touch, abrasion, or shear from strong airflow [15, 17]. When this occurs, the icephobicity of a SLIPS falls dramatically, and the porous nature of its substrate can cause ice adhesion to increase substantially due to the increased contact area available for ice attachment [17]. Performance failure may also be triggered by contaminants which either adhere or remove lubricant; because SLIPS rely on their lubricant to form moveable liquid layers which in turn cause ice to detach from the surface, contamination that alters the surface composition of this lubricant layer may cause the surface to lose icephobicity. Another source of surface oil depletion from SLIPS is from running water [14]. The flow of water (whether from a cleaning procedure or natural rainfall) can remove a significant amount of surface lubricant and affect their long term stability [18]. This can be problematic in places like the Canadian west coast, where there is not just snow, but precipitation year round, with rainfall on nearly half the days in a year [19]. Both problems can be potentially addressed by adopting a surface that is both icephobic and superhydrophobic.

Superhydrophobic surfaces minimize contact with water and often display a “self‐cleaning” effect, i.e., they can be rapidly and effectively cleaned by running water [20, 21, 22, 23]. This could mitigate accumulation of contaminants on SLIPS and therefore maintain its icephobicity for longer periods of time. It also can help delay the onset of ice formation through reducing dust available to serve as heterogeneous nucleation sites [24]. Superhydrophobicity can be achieved by combining low surface energy materials with surface morphology that is rough at the micro‐ and/or nanoscale, which has also been shown to retain lubricants more effectively for a SLIPS, i.e., reduce lubricant depletion [25]. However, SLIPS developed so far which are very hydrophobic are usually not very icephobic and vice versa. A material which is both superhydrophobic and icephobic could find applications in the marine industry, for example, increasing fuel efficiency and lifetime by reducing contact with water and preventing ice adhesion. In this paper, we describe a novel and facile fabrication method for icephobic and superhydrophobic SLIPS that have promise for improved longevity and performance over traditional SLIPS or superhydrophobic surfaces alone.

We have recently developed a protocol for fabricating superhydrophobic silicone films with water contact angles (WCAs) as high as 172 ± 1° and wetting hysteresis as low as 1° [20]. Briefly, acetone was used to crystallize a polycarbonate (PC) surface to generate a superhydrophobic substrate (WCA = 159 ± 1°) with hierarchical roughness. The surface is composed of microscale spherulites, which are covered by nanoscale tendrils of lamellae. The “rough” PC was then used as a master for nanocontact molding with PDMS, creating nanostructured, superhydrophobic PDMS [18]. The present work adapts this simple protocol to reproducibly create hierarchically structured PDMS films containing silicone oil that exhibit both ultrahigh water contact angle (> WCA 150°) and ultra‐low ice adhesion strength (IAS <20 kPa). The following sections will detail the successful fabrication and thorough characterization of our PDMS SLIPS that are both superhydrophobic and “supericephobic” (vide infra).

## 2 Results and Discussion

### 2.1 Icephobicity, Hydrophobicity, and Wetting Hysteresis: Morphological Optimization

Ice shedding capabilities are often measured using ice adhesion strength (IAS), which is usually expressed by units of kPa, with a smaller value indicating a lower shear stress (the force applied for a given cross‐sectional area) required to detach ice from a given surface. Thus, a material exhibiting lower IAS is more icephobic. In general, a surface is said to be icephobic when it exhibits an IAS of 100 kPa or lower. Values below 20 kPa are physically significant; it is the required threshold for ice to passively shed off a surface due to simple forces such as wind [2, 13]. Therefore, we propose the term “supericephobic” to describe materials exhibiting an IAS value of <20 kPa in analogy to superhydrophobic surfaces exhibiting a WCA > 150°.

**FIGURE 1:**
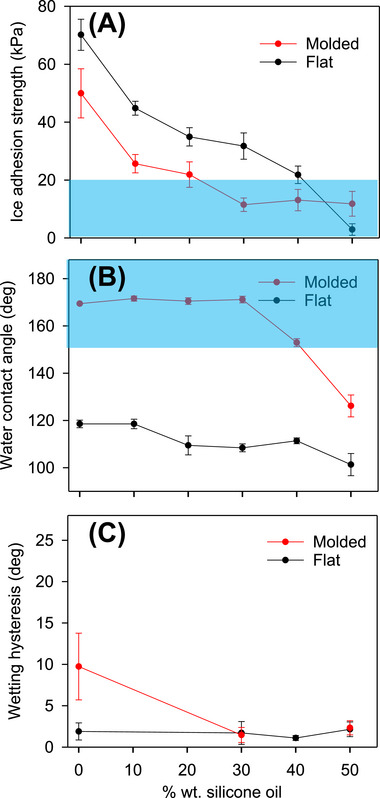
Ice adhesion strength (A), water contact angle (B), and wetting hysteresis (C) of flat and molded PDMS SLIPS with varied amounts of silicone oil. The error bars represent standard deviations obtained from at least three independently prepared samples. Shaded areas highlight the supericephobic (A) and superhydrophobic (B) “regimes”.

Figure 1A shows the dependence of IAS values of our fabricated PDMS SLIPS as function of the incorporated silicone oil in the precursor solution. The decrease of IAS is evident for both flat and molded (with micro‐ and nanostructured morphology from the crystalized PC template) samples upon addition of silicone oil (0–50 wt.% of the precursor solution) during PDMS curing. It is also apparent that molded (rough) samples show decreased IAS values compared to their flat (smooth) counterparts. This decrease in IAS compared to flat PDMS is no longer the case once the oil content reaches 40%; remarkablly it remains almost constant and clearly below 20 kPa. In fact, when the oil content reaches very high (i.e., 50%), the IAS value for a flat SLIPS is now lower than that of the molded sample. It should be noted that the anti‐icing performance of both flat and molded PDMS SLIPS is superior to the PC starting material, for which we measured IAS on the unmodified amorphous PC to be 123 ± 17 kPa.

WCAs were significantly different between smooth and molded PDMS samples as well, with the molded samples exhibiting much greater WCA values (Figure 1B), as expected of a “rougher” material with low surface energy. With no oil added, our rough PDMS samples showed superhydrophobicity (WCA = 169.4 ± 0.2°) compared to the 118.6 ± 1.6° of flat PDMS. As silicone oil content increases, WCA values begin to decrease for both flat and molded SLIPS. There are likely multiple reasons for this. For a hierarchically structured SLIPS, roughness namely at the nanoscale decreases with increasing oil content. Furthermore, the oil lowers the surface tension of the composite solid surface (primarily PDMS) and water would adhere to the surface to a greater extent.

Another important parameter for “superior” superhydrophobicity is wetting hysteresis—defined as the difference between the advancing and receding contact angles—which indicates the ease with which water droplets atop a surface begin to slide off, with lower values indicating lower tilt angles required [26]. Figure 1C depicts the wetting hysteresis determined for our PDMS SLIPS, which ranged from a high of 9.7 ± 4.0° for molded PDMS SLIPS (without adding silicone oil), to a low of 1.1 ± 0.3° (40% oil). There were no significant differences in wetting hysteresis between flat and molded PDMS samples other than for pure PDMS; in this case, we observed a significantly lower wetting hysteresis in flat samples compared to molded SLIPS. All other samples exhibited sliding angles lower than 3°.

**FIGURE 2:**
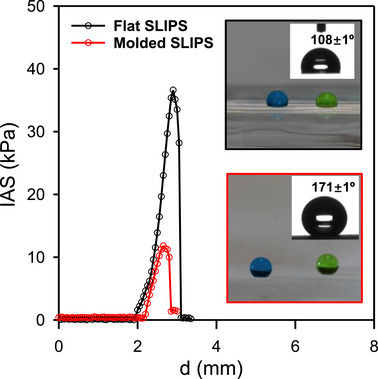
Representative force curves, i.e., plots of ice adhesion strength (IAS) vs. pushing distance (d) for flat (black curve) and molded (red curve) PDMS SLIPS (30% oil content). The inset pictures show colored water droplets on both surfaces with their corresponding contact angle measurements.

An important observation is that our SLIPS fabricated using 30% silicone oil is both supericephobic and superhydrophobic; as shown in Figure 2 it possesses an IAS of just 11.5 ± 2.3 kPa and a WCA of 171.2 ±1.5°. In comparison, the flat counterpart shows much higher IAS (31.7 ± 4.5 kPa) and lower water contact angles (108.5 ± 1.7)°, as such water droplets would be “beading up” on the molded sample but not on the flat SLIPS (inset pictures of Figure 2). Indeed, when a water droplet at the end of a needle were to bounce on the molded SLIPS, it would not adhere to the surface but return to the needle; in comparison the water droplet would detach from the needle and adhere to a flat SLIPS (see Figure S3 and Movie S1).

SLIPS exhibiting supericephobicity and superhydrophobicity simultaneously will undoubtedly have practical applications in a variety of environments; yet perhaps counterintuitively, this has been difficult to achieve because superhydrophobic surfaces are often not icephobic [27, 28]. The fact that surface nano‐/microstructures induce trapping of air pockets at the solid‐liquid interface leads to ultrahigh water contact angles and allows for water droplets to be easily rolled off [7, 29], however, ice or frost formation between the “cracks” caused by surface texture (i.e., voids between structures) sometimes can act as gripping points and increase IAS even further than on a flat surface [30, 31]. Moreover, when traditional superhydrophobic surfaces exhibit icephobicity, they typically only reduce IAS down to 50–100 kPa [13]. SLIPS have been demonstrated as an alternate, more consistent method of creating icephobic surfaces [13, 32].

Indeed, superhydrophobicity and icephobicity are related but distinct “concepts” both inspired by nature; lotus leaf‐inspired materials are often used for superhydrophobicity [22, 33, 34], while pitcher plants serve as inspiration for icephobic SLIPS [15, 35]. There have been numerous reports of both such materials, as well as more recently, some of switchable surfaces, i.e., the surface can be switched from repelling water through micro‐/nanostructures to a flatter slippery surface with infused lubricant [36, 37]. Nevertheless, this is the first instance of observing that both modes of repelling can be exhibited simultaneously, i.e., a micro‐/nanostructured SLIPS with ultrahigh WCA and extremely low IAS. In this case, we have essentially formed a functional “hybrid” of *Nepenthes* and *Nelumbo nucifera* plants to render excellent ice shedding capability and water repellency.

Despite having been the subject of much past research, for a long time, the relationship between icephobicity and superhydrophobicity had not been fully understood [28, 31, 38, 39, 40, 41]. In 2012, Nosonovsky and Hejazi elucidated the importance of the size of interfacial cracks, i.e., cracks or voids between the substrate and ice caused by surface structures [27]. If crack sizes are too small, a superhydrophobic surface will not be icephobic. IAS decreases with increasing crack sizes in general. We suggest that the hierarchical roughness of crystallized PC is the key factor that allows for our molded PDMS SLIPS with 30% oil to be both supericephobic and superhydrophobic; hierarchical roughness at the micro‐ and nanoscale allows for superhydrophobicity while wide interfacial “cracks” primarily at the microscale allow for supericephobicity.

**FIGURE 3:**
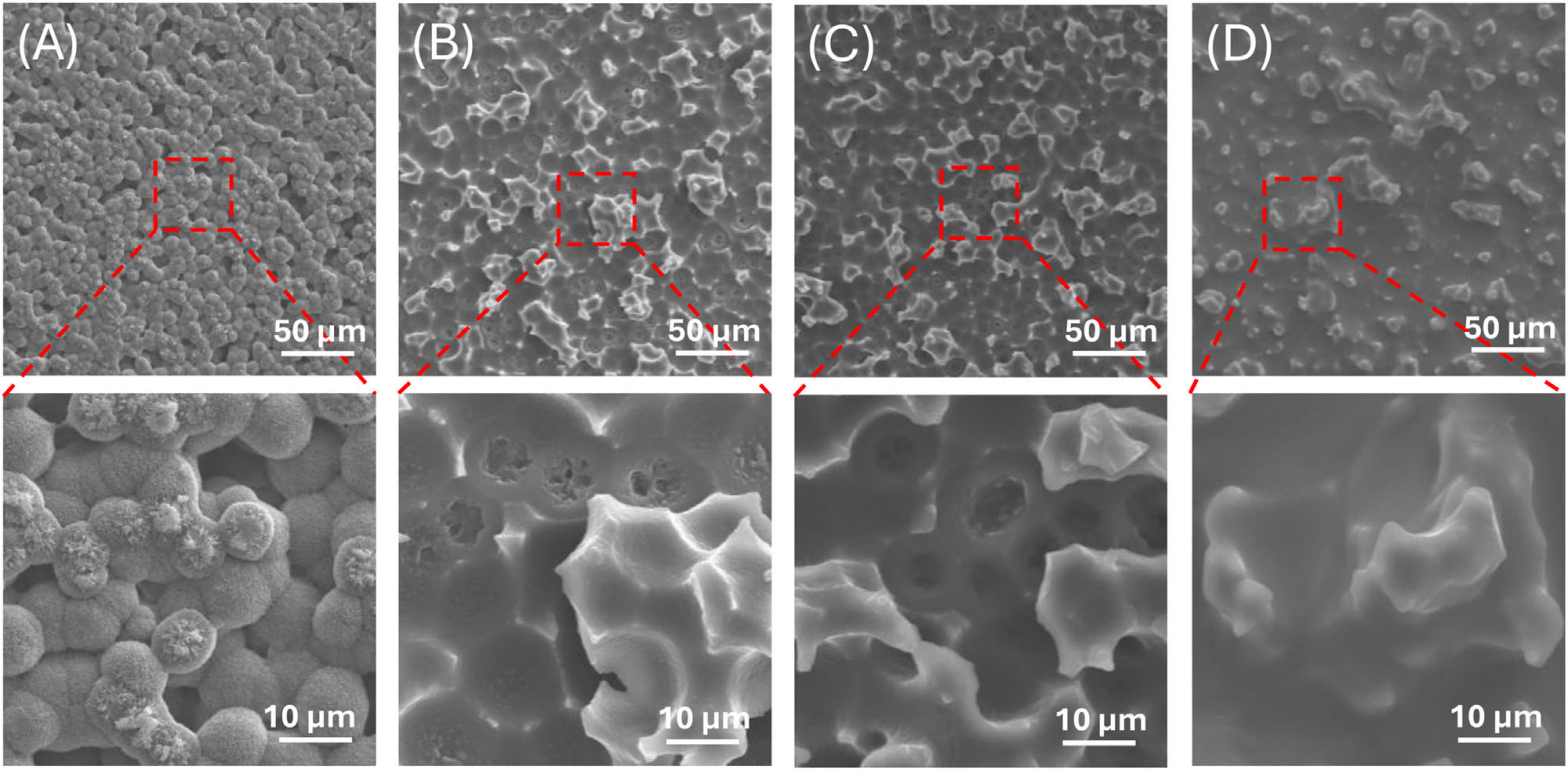
SEM images of crystallized PC template (A) and PDMS SLIPS made with 0% (B), 20% (C), and 30% (D) silicone oil; the bottom are corresponding zoom‐in images of the areas highlighted.

**FIGURE 4:**
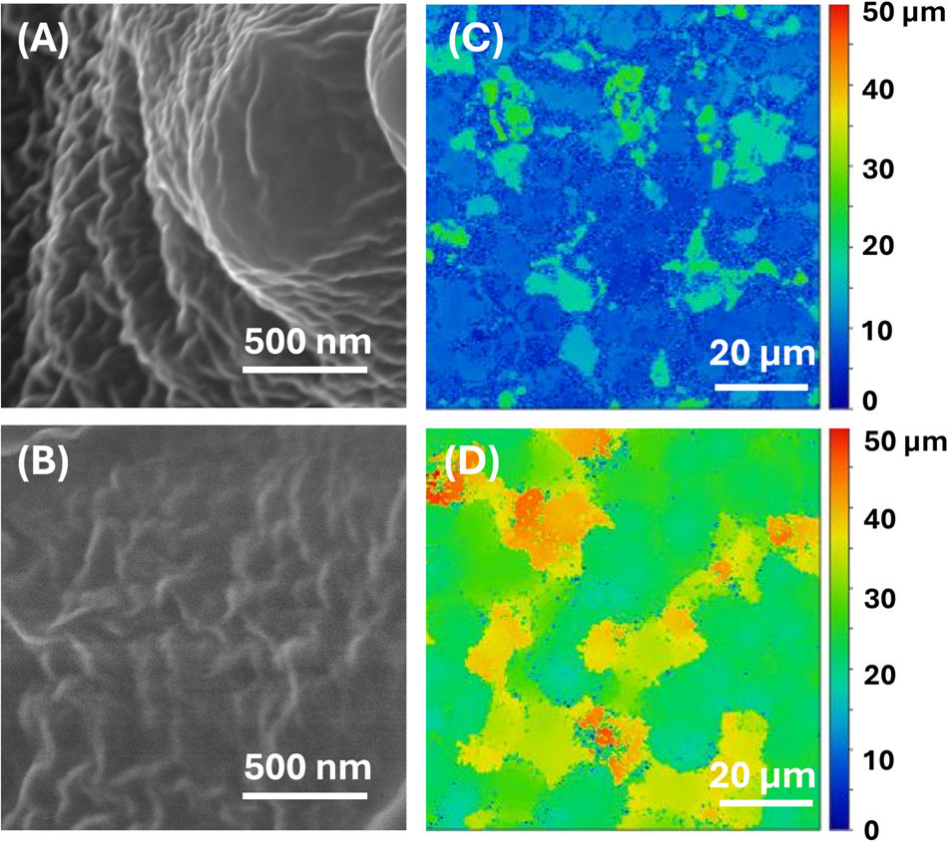
SEM and profilometry images of micro/nanoscale features of SLIPS fabricated with 30% (A,C) and 40% (B,D) silicone oil. Smoothening of nanofeatures is evident as oil content increases in both images (B,D).

SEM characterization of our PDMS SLIPS shown in Figure 3 confirms the hierarchical surface structures of our samples. Circular pits molded from the spherulites formed on the PC template are visible, leaving microscale “spikes”, from voids between spherulites as negatives across the entire surface (Figure 3A). As expected, molding with neat PDMS exhibits the highest molding resolution (Figure 3B), successfully molding pits of the top of spherulites and sharp spikes corresponding to the valleys between them. The “sharpness” of surface features decreases with increasing silicone oil content. At 20% oil, spherulite impressions become less defined and spikes become more rounded (Figure 3C); at 30% oil, spherulite impressions are even less prominent and the spikes become even more rounded (Figure 3D). Importantly, not all nanostructures are lost; while the spikes appear overall smoother, they still retain roughness at the nanoscale (Figure 4A). Under SEM, samples with 40% oil and beyond (for which the SLIPS are barely superhydrophobic) overall appear “fluffier”, and although nanoscale features are still present, they become rather smooth and flattened in comparison (Figure 4B); some nanoscale details are lost, and roughness is overall decreased on both the micro‐ and nanoscale. It is unclear whether this loss of feature sharpness is due to lubricant enveloping the structures, or if the PDMS matrix cannot maintain high resolution molding at higher oil percentages due to dilution of the “active” components for molding, i.e., the PDMS base and curing agent. However, even though these samples no longer exhibit superhydrophobicity, they still possess very low hysteresis (Figure 1C).

The profilometry images obtained on these samples provided further evidence to support the above hypothesis; while we can clearly observe nano‐ to micrometer scale roughness on the SLIPS of 30% oil (Figure 4C), such features become much less distinct on the surface with 40% oil (Figure 4D). In fact, the extracted roughness factors are different from each other; at 30% oil the sample showed an average roughness of R_a_ = 0.51 µm; at 40% oil, this lowers to 0.36 µm. At the same time, the R_q_ value (root mean square roughness) changes from 0.73 to 0.58 µm. These values support the WCA and IAS data gathered (Figure 1) and is in line with previous findings as discussed above, i.e., the surface roughness at both nanoscale and microscale significantly affect both hydrophobicity and icephobicity. When silicone oil content reaches 40%, however, it seems that a layer of lubricant may begin to engulf the surface (Figure 4D), and roughness measurements may not be entirely reliable. More in‐depth, mechanistic understanding of the correlation between the 3D nano‐ to micro structural features of PDMS SLIPS and their anti‐icing / water repelling properties certainly deserve further investigation, but beyond the scope of the present work.

### 2.2 Self‐Cleaning, Stability, and Durability of PDMS SLIPS: Performance Demonstration

**FIGURE 5:**
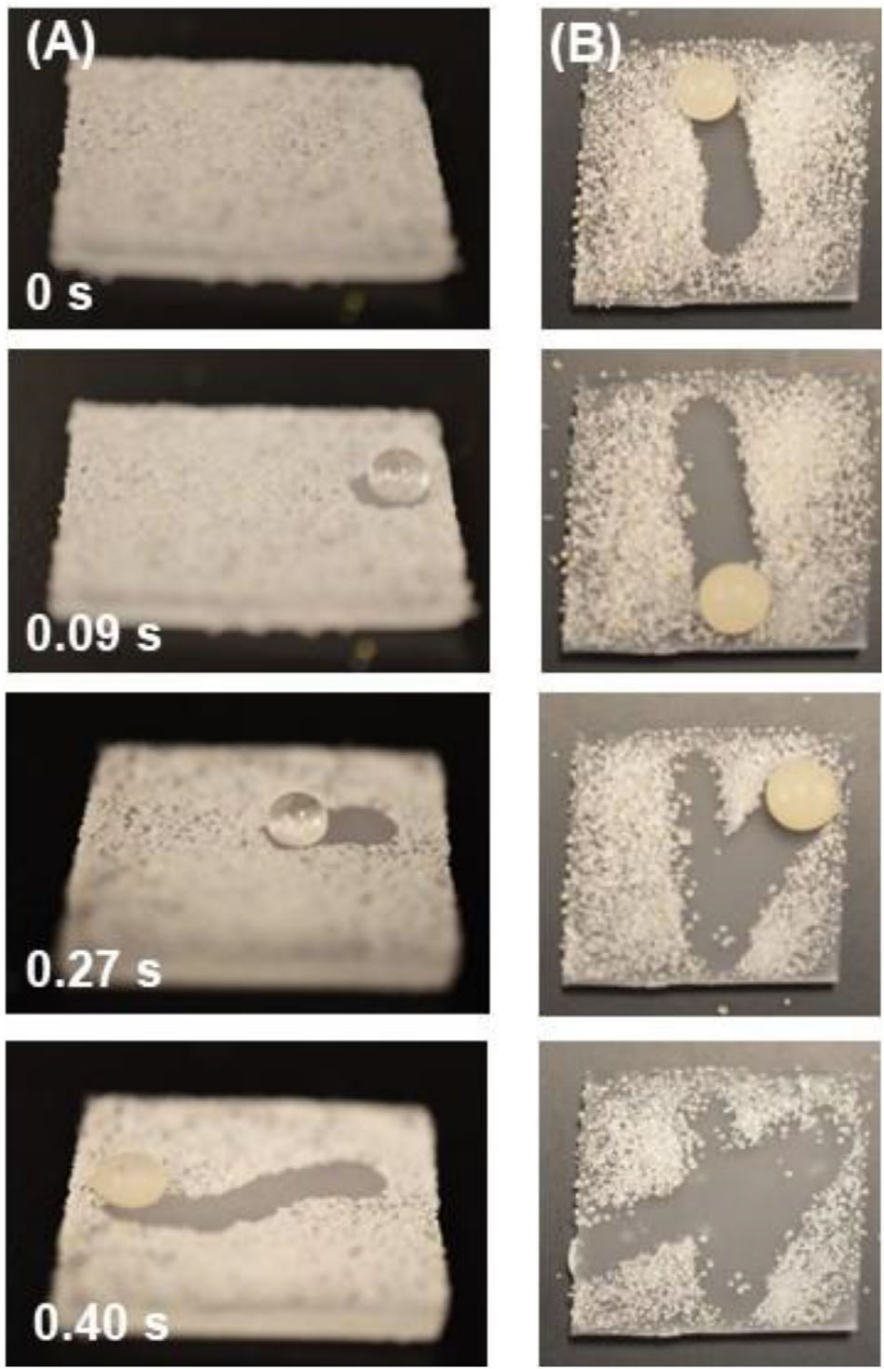
Demonstration of the “self‐cleaning” ability of our PDMS SLIPS. The surface is initially covered with sand, after which a water droplet is dispensed. The droplet readily rolls off the surface, removing surface contaminants. The two sets of pictures (A,B) are taken from two different angles. Also see Movie S2, which demonstrates the dynamic process of self‐cleaning of sand‐covered SLIPS.

As discussed above, the PDMS SLIPS (30% silicone oil) fabricated from molding crystallized PC is both superhydrophobic and supericephobic. It also exhibits a very low wetting hysteresis of 1.7 ± 1.4°. High WCAs and low wetting hysteresis values are associated with the so‐called self‐cleaning property, which describes the ability of rolling water droplets naturally formed by condensation or rainfall to clean the surface itself (popularly known as the lotus leaf effect) [23]. This self‐cleaning ability could be especially useful in maintaining the icephobicity by mitigating the collection of debris or contaminants on sample surfaces, allowing ease of cleaning and improving the longevity of these surfaces. As shown in Figure 5, contaminants such as sand can be easily removed by the motion of a water droplet deposited on the surface, i.e., sliding off the SLIPS while picking up the contaminants. This is due to both the water droplet and sand particles minimizing contact areas with the superhydrophobic SLIPS; the adhesion between the sand and water is greater than their adhesion to the surface. As the water droplet passes over the sand, it leaves behind a trail of clean surface.

**FIGURE 6:**
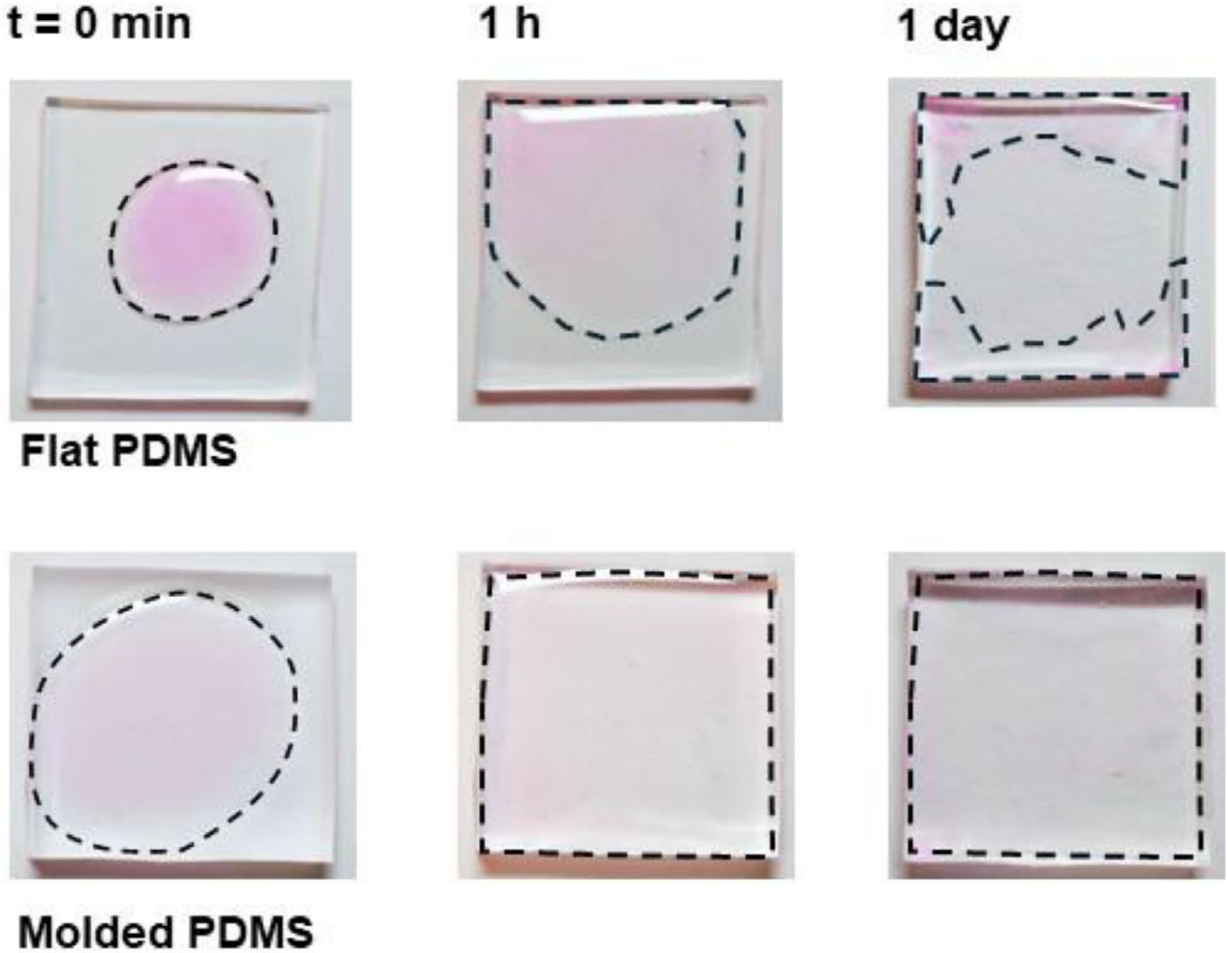
Spreading behaviour of a silicone oil droplet on flat PDMS and molded PDMS. A droplet of silicone oil (dyed with Rhodamine B to enhance visualization) was deposited onto each surface, and its spreading into a lubricating layer was monitored over time. The evolving spreading area is outlined in dashed black lines.

Another advantage of icephobic surfaces with hierarchical roughness of micro‐ and nanostructures is the greater retention of incorporated lubricants compared to flat surfaces [15, 25, 42]. Indeed, our molded PDMS SLIPS were able to form a uniform layer of silicone oil after placing a droplet on its surface in a short period of time (Figure 6); in contrast, on flat PDMS SLIPS, a drop of silicone oil does not spread into a uniform layer, i.e., remains in patches across the surface. This is more aligned with nature, as actual *Nepenthes* pitcher plants exhibit homogenous wetting with their lubricant [35]. It also illustrates longer term practical application of our molded SLIPS compared to flat SLIPS: the stronger affinity of the surface toward silicone oil indicates greater ease of replenishing the surface oil once the oil matrix is depleted, which has proven to be challenging with some other SLIPS [43].

To demonstrate lubricant stability in our PDMS SLIPS with oil content ≤ 30%, we gravimetrically examined any lubricant loss in our 30% oil samples, holding them upside down and allowing gravity to pull out any oil which may have phase separated. We measured the initial mass of samples and the mass after 60 days of inversion and any oil content loss to be virtually undetectable (∼0.01% mass difference). We also measured the amount of lubricant that could be removed from the surface by manually wiping with Kimwipes (Table S1) [42]. Wong et al. found that for a micro/nanostructured SLIPS, the structures help to retain the fused lubricant by “locking” it in place. On traditional superhydrophobic surfaces, once water permeates the cracks created by the micro‐/nanostructures, the water (and therefore, ice when frozen) can be locked into place due to adhesion with the material. With a hierarchically rough SLIPS, which retain oil, ice will be in contact with an intrinsically icephobic, fluid surface, preventing water from being “locked in”.

**FIGURE 7:**
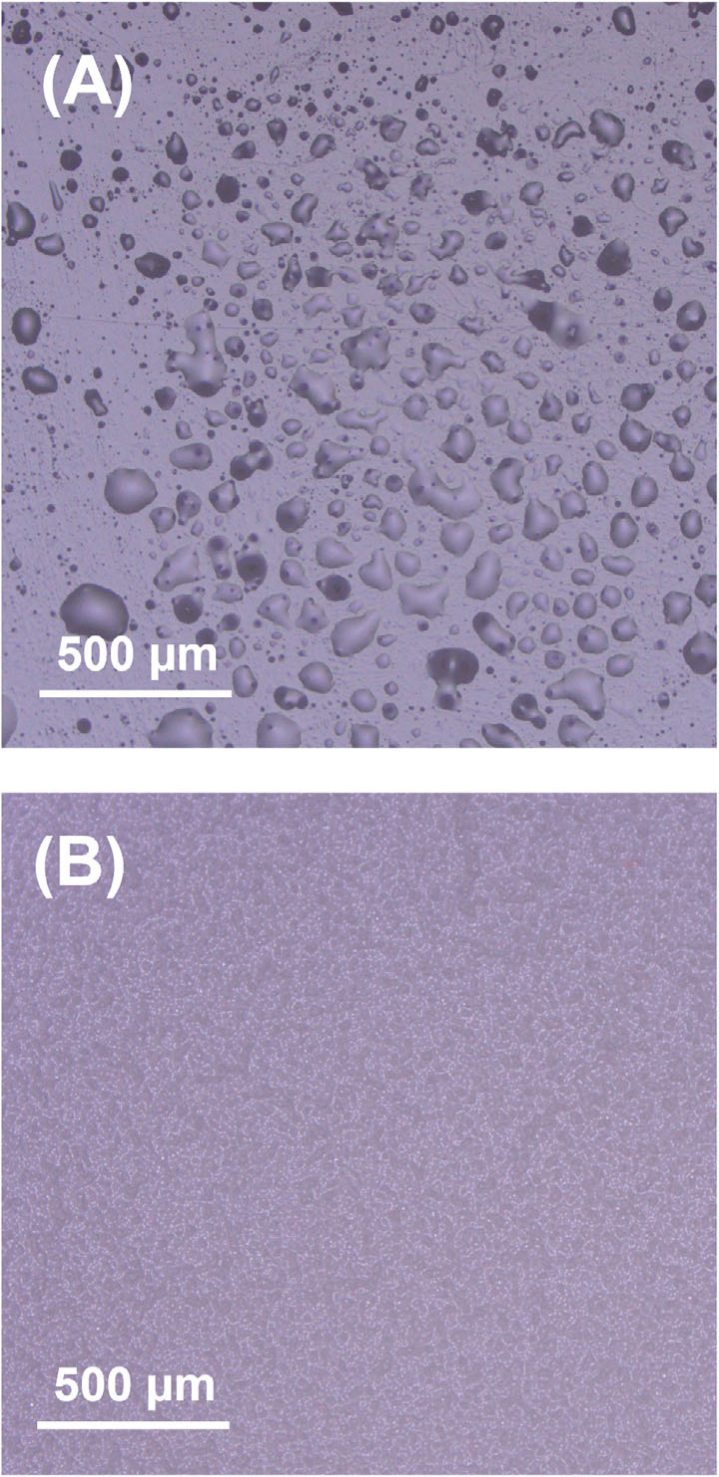
Optical microscope images of (A) a 40% oil flat PDMS SLIPS after three days and (B) 40% oil rough PDMS SLIPS after one week. Oil droplets are visible on the surface of the flat PDMS SLIPS while it is absent from the rough SLIPS.

The effect of micro/nanostructure increasing retention of lubricant is also evident by the lack of apparent phase separation of oil from our samples fabricated with at 40% oil when comparing to flat samples. Immediately after peeling from the mold after curing, both flat and rough samples show no easily visible oil on the surface of the SLIPS (although oil is evident if pressure is applied). However, after three days, visible oil appears on the surface of the flat sample as excess oil diffuses out from the matrix and coalesces, whereas this is not the case with rough samples. Even after several weeks, there is no oil on the surface of the rough samples. This is confirmed by visual inspection as well as optical microscopy imaging (Figure 7). This is even more evident from the changing IAS of flat PDMS SLIPS with ≥ 40% over time compared to that of molded samples; three days after peeling, the IAS of flat SLIPS decreases by 28% for 40% oil samples and by 48% for 50% oil samples, whereas IAS of molded samples remains stable. While a reduction in IAS is obviously desirable for increased icephobicity, being the result of oil phase separation from the matrix is not sustainable, and thus, a SLIPS which can retain oil better while still lowering IAS to the point of supericephobicity is an optimal balance of icephobicity and longevity.

As noted above, when oil content in the PDMS SLIPS samples exceeds 40%, oil begins to diffuse out. With oil content <40%, how then is IAS lowered in the absence of oil layer on the surface? This may occur through two mechanisms. First, IAS is affected by the modulus of the material. According to previous reports [2, 44], the stress (*τ*) involved in shearing a hard material from a soft one (e.g., ice from PDMS) is expressed by Equation (1).

(1) $$\tau =A_{\exp}\sqrt{W_{a}G/l}$$

where *A_exp_* is an experimental constant, *W_a_* is the work of adhesion, *G* is the shear modulus, and *l* is the thickness of the soft material. By adding silicone oil, even if there is no surface oil layer present, the PDMS becomes softer by nature of becoming more liquid‐like, lowering its modulus. This is consistent with the experimental observation that increasing silicone oil content in PDMS lowers its shear modulus. Moučka et al. found that for unmodified PDMS, *G* = 0.40 MPa, while *G* = 0.08 MPa with 30% silicone oil content (192‐cP) [45]. Second, although there is no visible oil layer present on the surface of these samples with lower silicone oil content, the oil adds uncrosslinked oligomeric chains of PDMS, which have mobility in the matrix, better enabling interfacial slippage. Note that PDMS without added oil still contains un‐crosslinked chains — albeit a small amount [46] — which can provide lubrication at the surface, influencing IAS and wetting behavior [47].

**FIGURE 8:**
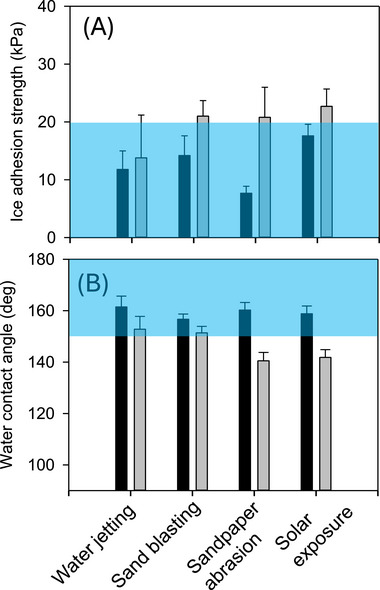
Durability tests of molded PDMS SLIPS (30% oil) by monitoring both ice adhesion strength (A) and water contact angle (B) changes. The black and gray bars represent the data before and after the test, respectively. Shaded areas are to highlight the superhdrophpobic (> 150°) and supericephobic (<20 pKa) regimes. Detailled experimental conditions are presented in the Supporting Information; Movie S3 shows how the water jetting and sand blasting experiments were performed.

To investigate the mechanical robustness of our SLIPS, we performed a series of standard tests, i.e., water jetting, sand blasting, sandpaper abrasion, and solar exposure following established literature procedures. As shown in Figure 8, the performance of the molded SLIPS with 30% oil content is quite impressive, i.e., they largely retained the anti‐icing and water‐repellent properties. We did observe discernible increase in the IAS and decrease of WCA values, especially with rather hush sandpaper abrasion and solar exposure experiments. These tests are intended to simulate the impact of weathering through rainfall, wind, and sunlight exposure. Moreover, we have also tested the robustness of the molded SLIPS by repeated icing and de‐icing cycles. Presented in the Supporting Information (Figure S8), we have confirmed that upon 15 cycles of icing and de‐icing procedure, the SLIPS being tested show no significant change in both the IAS and WCA values, i.e., retained its excellent anti‐icing and water‐repelling capabilities. It is believed that the flexible nature of PDMS and the hierarchical structure of the molded SLIPS can help to recover the structural integrity even if the surface layer undergoes degradation.

### 2.3 Composition Optimization and Environmental Sustainability

We have intentionally designed our SLIPS to be non‐fluorinated, as per‐ and polyfluoroalkyl substances (PFAS) have drawn much negative attention in the recent years for their numerous health and environmental concerns [48, 49, 50]. It is thus worth mentioning that we have not yet tried other types of lubricants, including fluorinated lubricants (which may further increase WCA and lower IAS) or even other types of silicone oils. A comparison of our SLIPS with some materials used in the literature for icephobicity, including those utilizing PFAS, is included in Table S2. The viscosity of the silicone oil used in this work was chosen to be 100‐cP after observing the IAS values of several PDMS samples conducted by Golovin et al.; for samples fabricated at a given weight composition of silicone oil and PDMS, the lowest IAS was observed for oil with 100‐cP viscosity [2]. Therefore, we opted to use the same type of silicone oil to fabricate our PDMS SLIPS. High affinity between the lubricant and the solid matrix is an important criterion for the design of SLIPS [32]. In fact, silicone oil is especially well‐suited for fabricating the PDMS‐based SLIPS because it simply resembles the oligomeric chains of PDMS. Because of this, there is exceptionally strong affinity between PDMS and silicone oil, leading to good miscibility and thus homogeneous distribution in the polymer matrix.

## 3 Conclusion

In this work, we have shown that properties of traditional superhydrophobic surfaces like high water contact angles (171.2 ± 1.5°) and low wetting hysteresis (1.7 ± 1.4°) can be combined with supericephobicity which allows passive shedding of ice off surfaces from minimal external forces like vibration and gravity (11.5 ± 2.3 kPa). This has been achieved by a simple bench‐top protocol of molding of hierarchically rough crystalline PC templates and systematically tuning the lubricant content to make nanosturctured PDMS SLIPS. This approach enables superior and more consistent icephobicity over traditional superhydrophobic surfaces, as well as better water repellency than traditional icephobic surfaces. Our study furthermore confirmed these PDMS SLIPS are self‐cleaning and stable over time. Alternatively, either superhydrophobicity or supericephobicity can be favored, with molded PDMS without oil (WCA > 150° and flat PDMS with ≥ 40% oil content (IAS ≤ 20) kPa. It may be noted that we did not examine freezing delay as an outcome parameter, as icephobicity and anti‐icing usually refer to different properties in the literature [34, 37, 51, 52, 53]; however, we acknowledge that this type of future work has fundamental utility impacts. Nonetheless, due to the potential of supericephobic and superhydrophobic surfaces in a variety of industrial applications, materials with a combination of these properties and increased durability from weathering, including from rainfall, wind, and sunshine exposure, represent a promising area of research and development. More importantly, this bench‐top fabrication approach can be readily scaled up for larger substrate materials and adapted to other polymeric (e.g., other types of elastomers), which augments its even broader practical promises.

## 4 Methods

### 4.1 Materials and Reagents

Sheets of Makrolon 2600 (M_w_ ≈ 26 000) polycarbonate (PC) were purchased from Bayer AG (Sheffield, AL). Acetone (99.8%) and methanol (99.8%) were purchased from Fisher Scientific (Hampton, NH) and Sigma–Aldrich (St. Louis, MO) respectively. Polydimethylsiloxane (PDMS) kits (Sylgard 184) consisting of an elastomer base and a curing agent were purchased from Ellsworth Adhesives (Germantown, WI). Deionized water (18.2 MΩ·cm) was procured from a Barnstead Easypure UV/UF water system from Thermo Scientific (Waltham, MA). 100‐cP silicone oil was purchased from Sigma–Aldrich (St. Louis, MO).

### 4.2 Fabrication of Polycarbonate Molds and PDMS SLIPS

Polycarbonate (PC) sheets were cut into 10 × 10 cm plates. The PC plates were washed with ethanol and deionized water and then left in an oven at 40° C to remove any moisture. Once dry and cooled to room temperature, they were immersed into an acetone bath for 120 s and then immediately immersed into a methanol nonsolvent bath for 120 s. Preparation and characterization of such PC molds are further detailed in our previous publication [20].

To fabricate the SLIPS, the elastomeric base and curing agent of PDMS kit were mixed 10:1 by weight percent as specified by the manufacturer. Silicone oil (100‐cP) was added with varied weight percentage, ranging from 0%–50% of the total weight. The 10:1 elastomeric base to curing agent ratio was maintained regardless of the amount of silicone oil incorporated. All compounds were mixed in a conical vial—first manually with a small spatula for at least 2 min, and then capped and mixed with a vortex mixer for another 2 min. The mixing procedure above was then repeated to ensure thorough mixing. Afterward, to ensure constant thickness, the SLIPS mixture was poured into a 1 mm thick mold consisting of a rubber spacer sandwiched by two silanized glass plates. In the case of hierarchically rough SLIPS, a sheet of crystallized PC was inserted as the master (template) (Figure S1). The setup was then placed into a vacuum chamber until visible bubbles disappear. Upon removal from the vacuum, the samples were allowed to cure for 48 h at room temperature, as this condition resulted in the greatest icephobicity; curing conditions of 24 h at 40°C and 35 min at 100°C were also tested (Figure S2). After curing, the resulting PDMS SLIPS were gently peeled off.

### 4.3 Characterization and Instrumentation

A custom apparatus was assembled for the purpose of determining ice adhesion strengh (IAS) on our PDMS samples, i.e., to measure the IAS value. Detailed descriptions to assemble and operate the device are presented in the Supporting Information (Figure S5). In brief, 300 µL of deionized water was pipetted into a cut centrifuge tube placed on top of a SLIPS. Once the ice had frozen, we used a motion stage (X‐LSM050A‐E03, Zaber Technologies) to push the digital force probe (Vernier) toward the ice column horizontally at a constant speed (0.05 mm/s), to produce a data set of applied force versus time. The IAS value was then calculated by dividing the maximum force needed to detach the ice column by the cross‐sectional area of the ice column based on the diameter of the column. Values are reported as averages from multiple measurements on at least five different samples for a given silicone oil weight percentage.

Scanning electron microscopy (SEM) images were obtained with an FEI Nova NanoSEM 430 SEM (Thermo Fisher Scientific, Hillsboro, OR) at an accelerating voltage of 5‐15 kV. Samples were coated with a 5−10 nm thick iridium layer using a Leica EM ACE600 Carbon & Iridium Coating System (Leica Microsystem Inc., Concord, ON) to improve surface conductivity. Profilometry imaging was performed using a Bruker Dektak XT (Billerica, MA) profilometer set to “map scan” mode; SLIPS was affixed to the sample stage using vacuum suction to prevent the sample from moving and distorting the scan.

Water contact angle measurements were taken using a VCA optima goniometer (AST Products Inc., Billerica, MA). A 1–2 µL droplet of deionized water was brought in contact with the surface by a syringe mounted on the goniometer. Both advancing and receding angles were determined by changing the volume of the water droplet using the syringe. These measurements were repeated at least three times with at least five independently prepared samples (flat and molded PDMS SLIPS, with and without silicone oil added).

To test the oil stability of our SLIPS, samples were adhered onto petri dishes. Weights of these samples were taken on an analytical balance, and the dishes were flipped upside down, remaining in this state for 60 days. Afterward, the weights were taken again to comopare.

The durability tests of the SLIPS followed established literature procedures; as detailed in the Supporting Information, water jetting and sand blasting experiments were performed at a height of 70 cm, with 100 mL of water or 100 g of sand, while the SLIPS sample was fixed on a tilt stage at 45°. We have used a P400 sandpaper for the abrasion tests, for which the SLIPS was dragged over a 30‐cm distance with a 60 g weight atop for 30 times. The solar exposure experiments were performed using a 2.2″ (5.7 cm) 300W UV Solar Simulator (Model 16S‐300‐2.2‐UV) from Solar Light Company, LLC (Glenside, PA) for 24‐h continued irradiation (290‐400 nm range).

### 4.4 Statistical Analysis

The numerical data presented were directly measured with the respective instruments (contact angle goniometer or force measurement setup) without any pre‐ or ‐post processing. The WCA and IAS values are presented as mean ± SD, with a sample size (n) of at least 3 (i.e., we have always prepared three or more replicates for each type of SLIPS sample). The contact angle values were obtained with the software provided by the manufacturer, and the roughness values for profilometry images were deduced using Gwyddion, for which its development is supported by the Department of Nanometrology, Czech Metrology Institute.

## Conflicts of Interest

The authors declare no conflicts of interest.

## Supporting information




**Supporting File 1**: advs74408‐sup‐0001‐SuppMat.pdf.

**Supporting File 2**: advs74408‐sup‐0002‐MovieS1.mp4.

**Supporting File 3**: advs74408‐sup‐0003‐MovieS2.mp4.

**Supporting File 4**: advs74408‐sup‐0004‐MovieS3.mp4.

## Data Availability

The data that support the findings of this study are available in the supplementary material of this article.
