# Supplementary material for: Combining the Pitcher and Lotus Plant: Supericephobic and Superhydrophobic Silicone Films
Source: Adv Sci (Weinh). 2026 Feb 21;13(23):e20538. doi: 10.1002/advs.202520538 (PMC13104152; doi:10.1002/advs.202520538)
Supplement: Supplementary file 1 — Supporting File 1: advs74408‐sup‐0001‐SuppMat.pdf. [file ADVS-13-e20538-s001.pdf]

Supporting Information

Combining the Pitcher and Lotus Plant: Supericephobic and Superhydrophobic Silicone Films

Cliff L. W. Ng,[†,§] Joshua Ham,[†,§] Sanpreet Kaur,[†] Clayton W. Schultz,[†] and Hua-Zhong Yu[†,‡,*]

[†]*Department of Chemistry and 4D Labs, Simon Fraser University, Burnaby, BC V5A 1S6, Canada*

[‡]*Department of Clinical Chemistry, Faculty of Allied Health Sciences, Chulalongkorn University, Bangkok 10330, Thailand*

[*] Corresponding author; e-mail: hogan_yu@sfu.ca

[§] These authors contributed equally to the work

This PDF file includes:

1. Preparation and Characterization of the Developed SLIPS……….……………………. S-2
2. Apparatus and Procedure for Measuring Ice Adhesion Strength (IAS) …………………. S-5
3. Durability Tests of the Developed SLIPS ……………………………………………….. S-6
4. Performance Comparison of the Developed SLIPS with Other Icephobic Films ……….. S-9

References ………………………………………………………………………………… S-10

Other Supplementary Materials:

- **Supplementary Movie 1.** Comparison of the dynamic wetting behavior of a water droplet contacting molded and flat SLIPS. Upon contact, the droplet collapses and wets the flat SLIPS surface, whereas on the molded SLIPS the droplet rebounds from the surface. indicating supressed wetting and reduced adhesion.

- **Supplementary Movie 2.** Self-cleaning demonstration of the SLIPS surface. Sand particles were deposited onto the sample, followed by exposure to water droplets, illustrating the readily removal of contaminants from the surface due to low adhesion and water repellency.

- **Supplementary Movie 3.** Sand blasting and water jetting tests performed on a SLIPS mounted on a glass slide. The sample was positioned on an inclined holder at 45° relative to the incoming flow. 100 g of sand or 100 mL was released from a height of 70 cm onto the sample.

1. Preparation and Characterization of the Developed SLIPS

Polydimethylsiloxane (PDMS) samples were prepared using a commercially available silicone elastomer kit (Sylgard 184, Ellsworth Adhesives), consisting of a vinyl-terminated PDMS base and a silicon hydride containing curing agent. The base and curing agent were mixed at a 10:1 weight ratio. For the SLIPS samples, silicone oil was added to the uncured PDMS mixture at the specified weight percentages prior to degassing. All mixtures were degassed under a vacuum to remove trapped air bubbles. The degassed PDMS or PDMS-silicone oil mixtures were cast onto pristine polycarbonate (PC) substrates or acetone-treated PC templates (**Figure S1**). Sample thickness was controlled using spacers of fixed thickness placed between the PC substrate and a silanized 10 × 10 cm^2 glass slide used as the top confinement plate. The glass slides were silanized to prevent adhesion between the cured PDMS and the glass surface. For PDMS samples without silicone oil, the PC substrate was removed from the casting assembly.

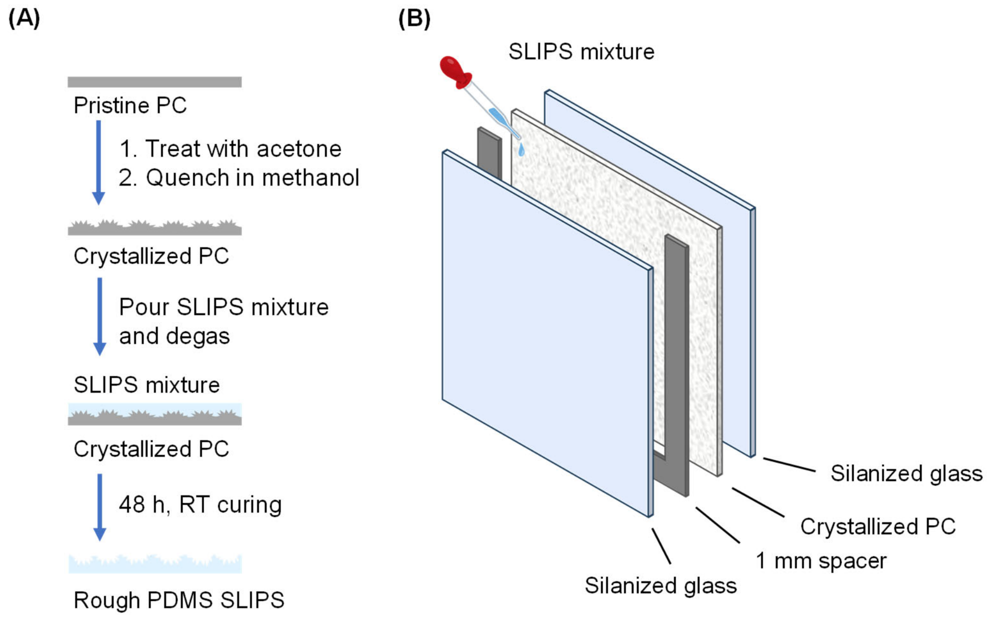

Figure S1. Preparation of PDMS SLIPS samples. (A) Schematic of molded PDMS SLIPS fabrication using an acetone-crystallized polycarbonate (PC) template. Pristine PC is first treated with acetone and subsequently quenched in methanol to induce surface crystallization. The SLIPS mixture is poured onto the crystallized PC and degassed under a vacuum. The PDMS SLIPS is typically cured at room temperature for 48 h before demoulding. (B) Schematic of the casting assembly to control the SLIPS thickness. The crystallized PC is sandwiched between two silanized glass slides using a 1-mm spacer (cut out from silicone mat for Crafts). The SLIPS mixture consists of PDMS base and crosslinker (10:1 ratio) with 0-50% silicone oil. Glass slides are pre-treated with chlorotrimethylsilane to reduce the adhesion and to facilitate the demoulding of PDMS SLIPS. Flat SLIPS samples are prepared without using the PC template.

We have systematically investigated the influence of curing temperature on mechanical properties and ice adhesion strength (IAS) of the fabricated SLIPS (**Figure S2**). It was found that curing at lower temperatures results in lower IAS values. Moučka et al. observed that at lower temperatures the PDMS material is softer and has lower shear modulus.[S1] Mechanistically, this observation is likely due to the platinum-based catalyst used in the hydrosilylation reaction between the vinyl group of the PDMS base and the Si-H bond containing crosslinker is more sluggish at low temperatures. Indeed, Moučka et al. quantitatively assessed the curing of PDMS to be faster at high temperatures, e.g., the rate constant, $k =$ 0.0079 s^{-1} at 100 °C and $k = 0.0209$ s^{-1} at 150 °C.

Based on this optimization study, curing at room temperature for 48 h was selected for all subsequent experiments, as it yielded the lowest IAS (**Figure S2**). For PDMS SLIPS, the presence of silicone oil further reduced the network stiffness by diluting the PDMS formulation and increasing chain mobility during curing, resulting in a softer elastomeric network.

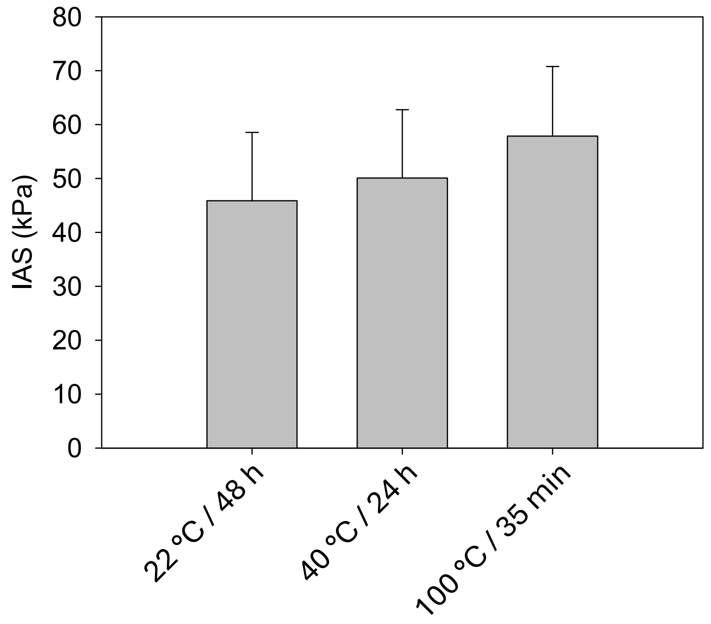

Figure S2. Ice adhesion strength of PDMS under various curing conditions. Error bars represent standard deviations determined from at least three replicated samples. The curing time was set by following the recommendation of the PDMS kit manufacturer.

Besides the characterization of PDMS SLIPS reported in the main text, we have performed additional experiments; for example, we have compared the dynamic wetting behavior of molded SLIPS and flat one by monitoring how a water droplet at the needle end to interact with the surface (**Figure S3** and **Supplementary Movie 1**). On the molded PDMS SLIPS surface, the droplet exhibits limited to no spreading upon contact and detaches rapidly from the surface (**Figure S3A**). In contrast, on the flat SLIPS surface, the droplet undergoes significant deformation upon contact and displays a stronger adhesion to the surface (**Figure S3B**).

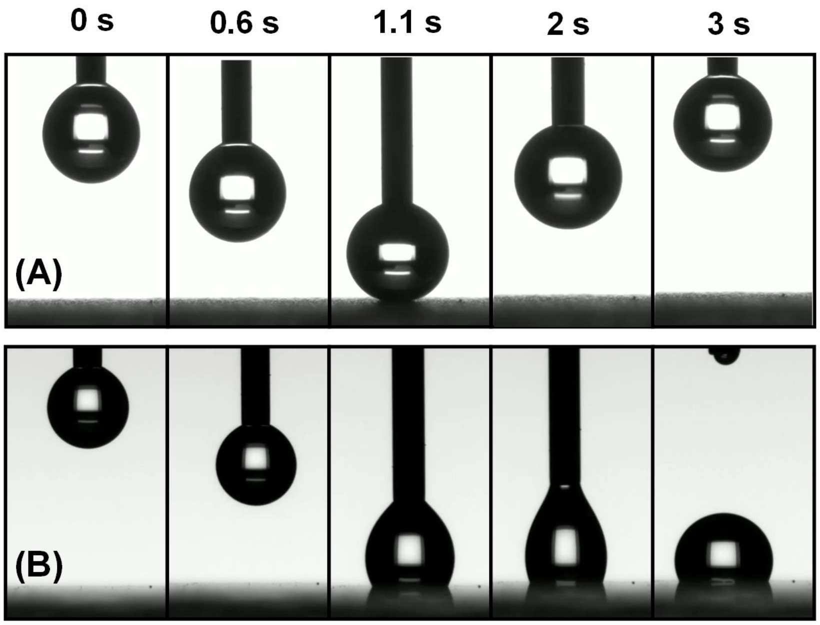

Figure S3. Time-resolved droplet-surface interactions on both molded (A) and flat (B) PDMS SLIPS (30% oil). Representative image sequences show the behavior of a water droplet when bouncing on a molded PDMS SLIPS surface, exhibiting minimal droplet pinning and adhesion (A), and on a flat PDMS SLIPS, showing increased droplet deformation and a stronger adhesion to the surface.

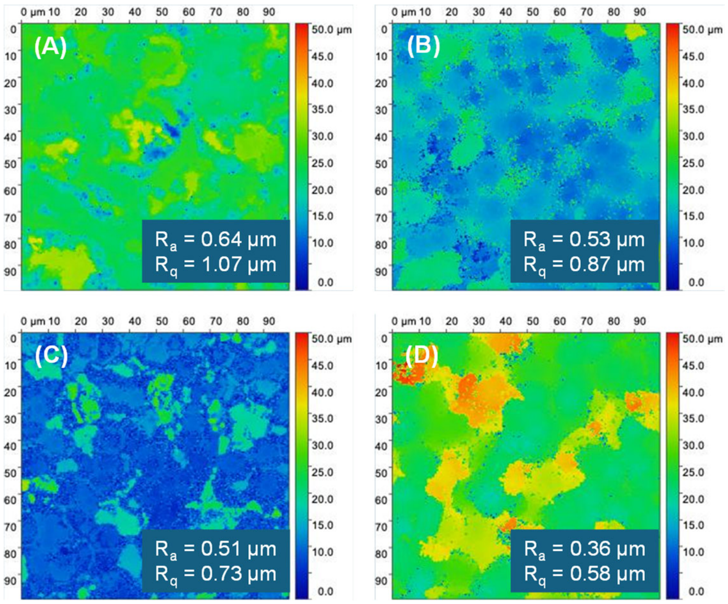

Figure S4. Profilometry images of molded PDMS samples with 0% (A), 20% (B), 30% (C), and 40% (D) silicone oil, showing the evolution of surface topography with increasing the oil content. The values of determined average roughness (R_a) and root-mean-square roughness (R_q) are listed as insets.

Surface topography of flat and molded PDMS samples with varying silicone oil contents was characterized using optical profilometry to visualize changes in surface morphology due to the oil infusion. Representative images of molded PDMS containing 0%, 20%, 30%, and 40% silicone oil are shown in **Figure S4**. In particular, all the SLIPS samples show heterogeneous surface features, with microscale height variations becoming less pronounced as the oil content increases. The average roughness (R_a) and root-mean-square roughness (R_q) values extracted from the profilometry data are list as insets in **Figure S4.** The results are also described side-by-side with SEM images in the main text (**Figure 4**).

2. Apparatus and Procedure for Measuring Ice Adhesion Strength (IAS)

As depicted in **Figure S5**, the custom-built force measurement setup is consists of three main components: a motion stage (motorized linear actuator, X-LSM050A-E03, Zaber Technologies), a force probe (Go Direct Force and Acceleration Sensor, Vernier Canada), and a Peltier plate (TEC1-12706, Generic, Amazon Canada). The force probe is attached onto the motion stage using a threaded rod. The Peltier plate rests on a heat sink and fan (from an AMD CPU), which is connected to a 12 V power supply. This is necessary to cool the hot side of the Peltier plate, which otherwise heats up rapidly. These components are bolted down onto a metal plate to immobilize all components and prevent loss of force due to sliding. A digital thermostat wired to the Peltier plate allows accurate control of the temperature, which is connected to another 12 V power supply.

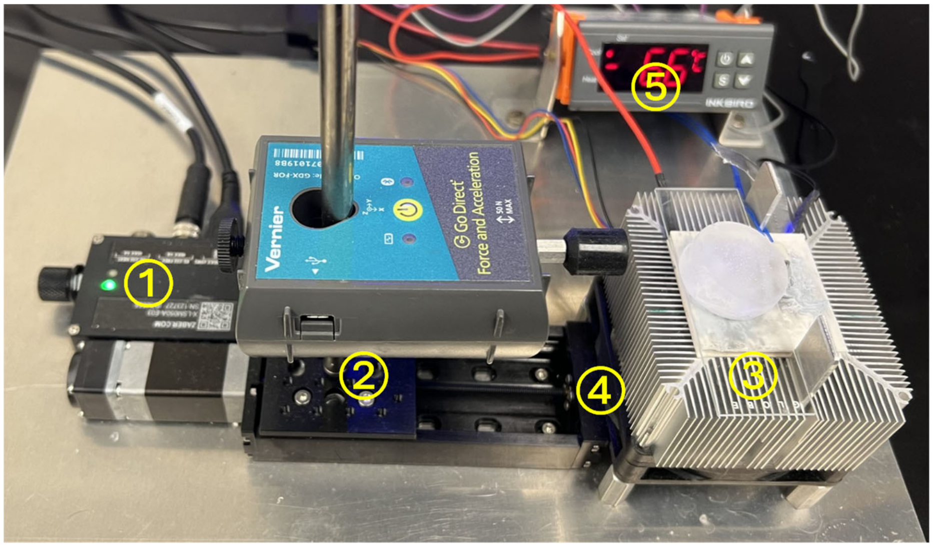

Figure S5. Custom-built apparatus for testing ice adhesion strength. (1) motion stage (motorized linear actuator), (2) force probe, (3) Ice sample/SLIPS on the Peltier plate, (4) cooling fan with heat sink, and (5) digital thermometer.

Data from the force probe (Bluetooth connection) is gathered using Vernier Graphical Analysis software. The temperature of the Peltier plate was verified as -12 ± 2 °C using an infrared thermometer prior to sample freezing. Samples were mounted on a glass slide and placed onto the Peltier plate using

thermal paste to enhance thermal conductivity. The slides rest against the attached backstop to prevent slippage. A small petri dish cover is used to cover the sample to reduce the amount of frost formation. Measurements were performed at ambient temperature and humidity, with an average air temperature of 22–25 °C and relative humidity of ~35%.

Ice columns were formed directly on the PDMS SLIPS samples. The force probe was positioned approximately 1 mm away from the base of the ice column, and the stage advanced at a constant speed of 0.05 mm/s until ice detachment occurred. Ice adhesion strength (IAS) was calculated by dividing the maximum recorded force (F) by the cross-sectional area of the ice column (A). A typical force recorded (Force vs. time) is shown in **Figure S6**.

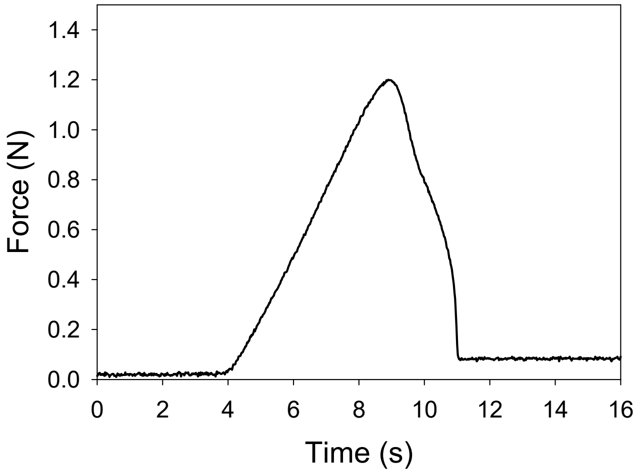

Figure S6. A representative force-time curve obtained using our custom-built setup and the associated software. Data depicted is for SLIPS with 30% silicone oil (fabricated from molding PC template that was crystallized by acetone for 2 min followed by immersion into a methanol bath for 2 min). As the contact area (A, cross section at the bottom) of the ice pillar and the moving speed (mm/s) of the linear actuator is precisely controlled, so that we can obtain the IAS value from the ratio of measured force (N) and the ice/SLIPS contact area (mm^2) and plotted with respect to the moving distance (d), shown in Figure S8 (inset) and Figures 1-2 in the main text and.

3. Durability Tests of the Developed SLIPS

The mechanical robustness / durability of the PDMS SLIPS was thoroughly evaluated using water jetting, sand blasting, sandpaper abrasion, solar exposure, and repeated icing/de-icing experiments. These tests were selected to probe the resistance of the SLIPS surfaces to mechanical and environmental stresses using test configurations commonly adapted in previous studies of superhydrophobic and icephobic materials.[S2-S5] While no accepted standard exists for these durability tests, experimental parameters were independently chosen and held constant to ensure internal consistency and reproducibility across all measurements. Experimental setups of the water jetting, sand blasting, and sandpaper abrasion are shown in **Figure S7**.

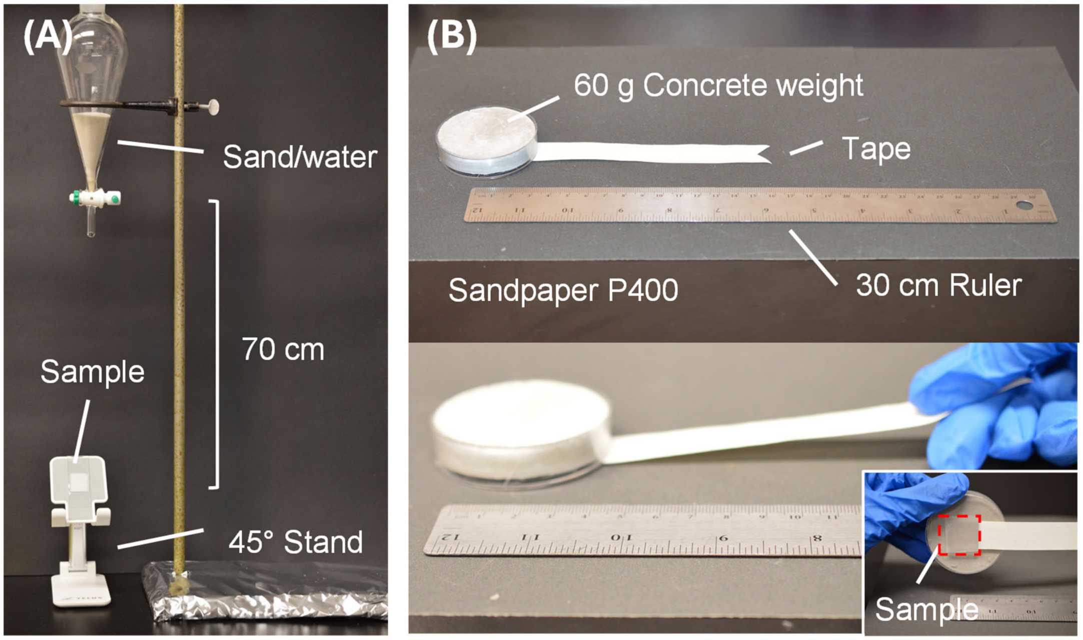

Figure S7. Experimental setups used for mechanical durability testing of PDMS SLIPS. (A) Gravity-driven water jetting and sand blasting setup. A separatory funnel positioned 70 cm above the sample delivers either deionized water or sand onto the PDMS SLIPS mounted on a holder tilted at 45°. (B) The setup for sandpaper abrasion tests. The PDMS SLIPS sample is placed face-down on P400 grit sandpaper and subjected to linear abrasion under a constant weight applied by a 60-g concrete block. The sample is pulled linearly along a 30 cm track using an adhesive tape.

As shown in **Figure S7A**, water jetting and sand blasting tests were conducted using a gravity-driven setup adapted from previously reported durability tests.[S2-S3] PDMS SLIPS samples were mounted on a rigid holder and tilted at 45° relative to the incoming flow. A separatory funnel positioned 70 cm above the sample surface was used to deliver either deionized water or sand onto the surface. For water jetting tests, 100 mL of deionized water was released from the funnel, producing a 7.1 mL/s continuous stream that hit the SLIPS under gravity. For sand blasting tests, 100 g of sand, particle size 30-100 mesh, was released from the same height, resulting in a 1.2 g/s continuous impact to the sample surface. These tests were designed to impose combined normal and shear stresses representative of environmental exposure such as rainfall, erosion, and wind. After testing, samples were gently cleaned to remove residual debris and subsequently characterized to evaluate the retention of superhydrophobic and supericephobic properties. As shown in **Figure 8** of the main text, there are discernible changes in both WCA and IAS values after these tests, though the SLIPS sample largely retained its anti-icing and water repellency properties (detailed discussions see the main text).

Sandpaper abrasion tests were performed to assess resistance to mechanical wear using a protocol adapted from commonly used linear abrasion methods.[S4] As depicted in **Figure S7B**, PDMS SLIPS samples were placed face-down on P400-grit sandpaper fixed to a flat substrate. A 60 g concrete weight was placed on top of the sample to apply a constant normal weight. The sample was then pulled linearly

across the sandpaper over 30 cm at a constant speed using adhesive tape. One abrasion cycle was defined as a single forward pass along the abrasion track, and a total of 30 cycles were performed. Samples were then characterized after abrasion to evaluate changes in surface wetting and icephobic properties. Compared to water jetting and sand blasting, sandpaper abrasion resulted in a more pronounced decrease in WCA and an increase in IAS, with values approaching the boundaries of superhydrophobicity and supericephobicity.

Solar exposure tests were performed using a protocol similar to those previously used to assess UV stability of lubricated surfaces.[S5] PDMS SLIPS samples were exposed to continuous UV irradiation for 24 h using a 2.2" (5.7 cm) 300 W UV solar simulator (Model 16S-300-2.2-UV, Solar Light Company Ltd), producing a UV output in the 290-400 nm range. Before and after the exposure, samples were characterized by both WCA and IAS measurements. While icephobic behaviour was largely retained, WCAs were reduced to ~ 140 ° (**Figure 8** in the main text). Notably, the WCA recovered to values comparable to the initial measurements after two days of storage under ambient conditions following the solar exposure.

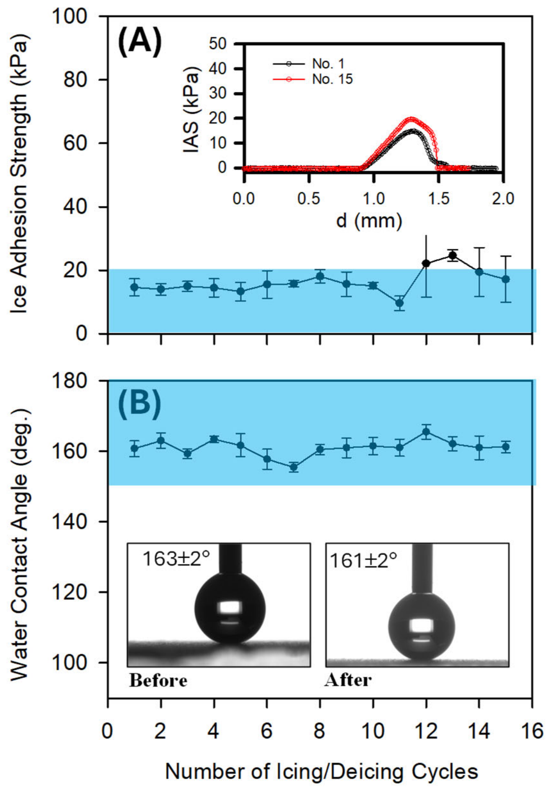

Figure S8. Durability tests of molded PDMS SLIPS by repeating the formation and removal of ice pillars, and monitoring both ice adhesion strength (IAS) and water contact angle (WCA) variations after each cycle. The insets show the first (No. 1) and the last (No. 15) IAS curves and water contact angles (before and after all 15 cycles). The shaded areas in both plots highlight the superhydrophobic and supericephobic "regimes".

Icing and de-icing durability tests were conducted to evaluate the stability of the PDMS SLIPS under repeated ice formation and removal using procedures reported by Barthwal et al.[S6] Ice pillars were formed directly on the sample surfaces using deionized water on a temperature-controlled Peltier plate maintained at -12 °C. IAS was measured by lateral shear using the custom apparatus described in **Figure S5**. After each icing/de-icing cycle, residual water was removed, and the sample was allowed to equilibrate to room temperature before characterization. WCAs were measured after each cycle, and this process was repeated for 15 cycles. As shown in **Figure S8,** repeated icing/de-icing led to insignificant changes in IAS and WCA values (within the experimental uncertainties), indicating their least disruption of the lubricated layer and surface structure, i.e., the SLIPS retained excellent superhydrophobicity and supericephobicity after multiple icing/de-icing cycles.

As part of the oil retention tests (discussed in the main text), for the wiping experiments, oil was removed by placing a Kimwipe™ tissue onto the surface of the samples (on the hierarchically rough side in the case of the molded sample) and allowing the wipe to become saturated before being removed (Table S1). This represents a more active removal of surface oil rather than a passive setting of just gravity. Only the weight of the wipe was used; no additional downward force was applied. A wipe cycle consisted of a single complete wipe of the sample surface and a subsequent wait time of 1 h. No significant changes in % weight reductions were found using independent samples *t*-tests comparing molded and flat PDMS samples ($p = 0.17$).

Table S1. The % weight reduction in flat vs. molded PDMS SLIPS (50% silicone oil) after two wipe cycles with a Kimwipe™. Error bars represent the standard deviation determined from three independent experiments.

	**% change 1 wipe**	**% change 2 wipes**	**Overall % change**
Flat SLIPS	-0.75 ± 0.07	-0.53 ± 0.23	-1.27 ± 0.29
Molded SLIPS	-0.58 ± 0.25	-0.12 ± 0.18	-0.71 ± 0.22

4. Performance Comparison of the Developed SLIPS with Other Icephobic Films

Table S2. Comparison with PDMS or other polymeric materials from landmark papers in terms of icephobicity and hydrophobicity. Blue shading indicates superhydrophobicity; orange shading indicates supericephobicity, and yellow shading indicates PFAS-based materials.

**Ref.**	**Matrix material**	**Type of lubricant**	**Roughness**	**Hardness**	**WCA (deg)**	**IAS (kPa)**
This work	PDMS	Silicone oil	Micro, nano	Elastomeric	171.2 ±1.5	11.5 ± 2.3
S6	PDMS coated on Al	Silicone oil	Micro with nanopores	Elastomeric on hard substrate	104	22 ± 5
S7	PDMS	Silicone oil	Micro	Elastomeric	155	38†

S8	Perfluoroalkyl functionalized polypyrrole on Al	Perfluoro-polyether	Micro, nano	Hard	117 ± 3*	15.6 ± 3.6
S9	VytaFlex40 polyurethane	Cod liver oil	Flat	Elastomeric	43*	4†
	PDMS	None??	Micro	Elastomeric	165*	26 ± 3†
S10	Branched PDMS crosslinked by functionalized POSS on stainless steel	Silicone oil	Flat	Hard	104.6-108.3	9.1-22.2
S11	PDMS coated on Al	Silicone oil	Nano	Elastomeric on hard substrate	120.5	50
S12	80/20 PEMA/fluoro decyl POSS on steel	None	Flat	Hard	123.8 ± 1.2*	165 ± 27†
S13	PTES coated on Al	None	Micro, nano	Hard	164	220

* Advancing contact angle reported in lieu of static contact angle.
† Humidity not stated. Thus, it should be assumed that these measurements took place in low-humidity conditions, and the same IAS may not translate to a real-world environment. For a superhydrophobic surface, the absence of humidity significantly increases its icephobicity (lowers IAS).
POSS: polyoctahedral silsesquioxanes, PEMA: polyethylmethacrylate, PTES: 1H,1H,2H,2H-perfluorodecyltri-ethoxysilane.

References

(S1) Moučka, R.; Sedlačík, M.; Osička, J.; Pata, V. Mechanical Properties of Bulk Sylgard 184 and Its Extension with Silicone Oil. *Scientific Report* **2021**, *11* (1), 19090. https://doi.org/10.1038/s41598-021-98694-2.

(S2) Wang, N.; Xiong, D.; Deng, Y.; Shi, Y.; Wang, K. Mechanically Robust Superhydrophobic Steel Surface with Anti-Icing, UV-Durability, and Corrosion Resistance Properties. *ACS Applied Materials & Interfaces* **2015**, *7* (11), 6260–6272. https://doi.org/10.1021/acsami.5b00558.

(S3) Ibáñez-Ibáñez, P. F.; Javier, F.; Cabrerizo-Vílchez, M. A.; Rodríguez-Valverde, M. A. Mechanical Durability of Low Ice Adhesion Polydimethylsiloxane Surfaces. *ACS Omega* **2022**, *7* (24), 20741–20749. https://doi.org/10.1021/acsomega.2c01134.

(S4) Pan, S.; Chen, M.; Wu, L. Fabrication of a Flexible Transparent Superomniphobic Polydimethylsiloxane Surface with a Micropillar Array. *RSC Advances* **2019**, *9* (45), 26165–26171.

(S5) Eleni, P. N.; Krokida, M. K.; Polyzois, G. L. The Effect of Artificial Accelerated Weathering on the Mechanical Properties of Maxillofacial Polymers PDMS and CPE. *Biomedical Materials* **2009**, *4*(3), 035001. https://doi.org/10.1088/1748-6041/4/3/035001.

(S6) Barthwal, S.; Lee, B.; Lim, S.-H. Fabrication of Robust and Durable Slippery Anti-Icing Coating on Textured Superhydrophobic Aluminum Surfaces with Infused Silicone Oil. *Applied Surface Science* **2019**, *496*, 143677. https://doi.org/10.1016/j.apsusc.2019.143677.

(S7) Yong Han Yeong; Wang, C.; Wynne, K. J.; Gupta, M. C. Oil-Infused Superhydrophobic Silicone Material for Low Ice Adhesion with Long-Term Infusion Stability. *ACS Applied Materials & Interfaces* **2016**, *8*(46), 32050–32059. https://doi.org/10.1021/acsami.6b11184.

(S8) Kim, P.; Wong, T.-S.; Alvarenga, J.; Kreder, M. J.; Adorno-Martinez, W. E.; Aizenberg, J. Liquid-Infused Nanostructured Surfaces with Extreme Anti-Ice and Anti-Frost Performance. *ACS Nano* **2012**, *6*(8), 6569–6577. https://doi.org/10.1021/nn302310q.

(S9) Golovin, K.; Kobaku, S. P. R.; Lee, D. H.; DiLoreto, E. T.; Mabry, J. M.; Tuteja, A. Designing Durable Icephobic Surfaces. *Science Advances* **2016**, *2* (3), e1501496. https://doi.org/10.1126/sciadv.1501496.

(S10) Gao, S.; Liu, B.; Peng, J.; Zhu, K.; Zhao, Y.; Li, X.; Yuan, X. Icephobic Durability of Branched PDMS Slippage Coatings Co-Cross-Linked by Functionalized POSS. *ACS Applied Materials & Interfaces* **2019**, *11* (4), 4654–4666. https://doi.org/10.1021/acsami.8b19666.

(S11) Zhu, L.; Xue, J.; Wang, Y.; Chen, Q.; Ding, J.; Wang, Q. Ice-Phobic Coatings Based on Silicon-Oil-Infused Polydimethylsiloxane. *ACS Applied Materials & Interfaces* **2013**, *5* (10), 4053–4062. https://doi.org/10.1021/am400704z.

(S12) Meuler, A. J.; Smith, J. D.; Varanasi, K. K.; Mabry, J. M.; McKinley, G. H.; Cohen, R. E. Relationships between Water Wettability and Ice Adhesion. *ACS Applied Materials & Interfaces* **2010**, *2* (11), 3100–3110. https://doi.org/10.1021/am1006035.

(S13) Wang, Y.; Xue, J.; Wang, Q.; Chen, Q.; Ding, J. Verification of Icephobic/Anti-Icing Properties of a Superhydrophobic Surface. *ACS Applied Materials & Interfaces* **2013**, *5* (8), 3370–3381. https://doi.org/10.1021/am400429q.
